# The ADNP Syndrome and CP201 (NAP) Potential and Hope

**DOI:** 10.3389/fneur.2020.608444

**Published:** 2020-11-24

**Authors:** Illana Gozes

**Affiliations:** The Elton Laboratory for Molecular Neuroendocrinology, Department of Human Molecular Genetics and Biochemistry, Sackler Faculty of Medicine, Sagol School of Neuroscience and Adams Super Center for Brain Studies, Tel Aviv University, Tel Aviv, Israel

**Keywords:** ADNP, ADNP syndrome, CP201 (NAP, davunetide), microtubules (MT), *Adnp*^+/−^ mice, tau

## Abstract

Activity-dependent neuroprotective protein (*ADNP*) syndrome, also known as Helsmoortel-Van Der Aa syndrome, is a rare condition, which is diagnosed in children exhibiting signs of autism. Specifically, the disease is suspected when a child is suffering from developmental delay and/or intellectual disability. The syndrome occurs when one of the two copies of the *ADNP* gene carries a pathogenic sequence variant, mostly a *de novo* mutation resulting in loss of normal functions. Original data showed that *Adnp*^+/−^ mice suffer from learning and memory deficiencies, muscle weakness, and communication problems. Further studies showed that the ADNP microtubule-interacting fragment NAP (called here CP201) resolves, in part, *Adnp* deficiencies and protects against *ADNP* pathogenic sequence variant abnormalities. With a clean toxicology and positive human adult experience, CP201 is planned for future clinical trials in the *ADNP* syndrome.

## Background

The *ADNP* syndrome (https://www.orpha.net/consor/cgi-bin/OC_Exp.php?Lng=EN&Expert=404448; https://rarediseases.info.nih.gov/diseases/12931/adnp-syndrome) traits include limitations of social interactions and communication along with stereotypic, repetitive behavior, and restricted interest (1). *ADNP de novo* mutations (pathogenic sequence variants) causing syndromic autism were first described by O'Roak et al. and later extended by Helsmoortel et al. as reviewed in the laboratory of Illana Gozes, the discoverer of the *ADNP* gene (2–8).

The human *ADNP* gene is ~40 kilobases long and contains five exons and four introns (5). The gene is located on the q13.13 band of chromosome 20 (8, 9). The protein comprises 1,102 amino acids including asparagine–alanine–proline–valine–serine–isoleucine–proline–glutamine (NAPVSIPQ), which is an 8-amino-acid neuroprotective peptide called NAP (also discovered by the Gozes Laboratory). NAP is referred here to as CP201 (1, 4, 7, 8).

The *ADNP* gene is one of the most prevalent single mutated genes within the autism spectrum disorders (ASDs) (5, 6, 10, 11). According to the original description, the *ADNP* syndrome is estimated to account for 0.17% of all cases of ASD (4, 12).

More than 400 genes are regulated by ADNP, which are critical for brain formation, organ development, cognition, and motor function (6, 13–16). In the nucleus, ADNP is a member of a chromatin remodeling complex that is responsible for RNA transcription and splicing (13, 17–19). In the cytoplasm, ADNP has been shown to correlate with the microtubule (MT)–associated protein Tau, leading to dynamic Tau expression and protection against Tau pathology (hyperphosphorylation) (20). Tau hyperphosphorylation has been associated with neurodegeneration along with cognitive decline (6, 20, 21). Importantly, ADNP interacts directly with the MT end-binding proteins (EB1 and EB3). When there is a mutation and one of the *ADNP* alleles is lost (or dysfunctional), there is a disruption in the MT–EB protein interaction (6). This causes a negative impact on brain formation leading to decreased learning skills and memory (5).

The syndrome occurs when one of the two copies of the *ADNP* gene is mutated and loses its normal function (4). The mutation is most often a *de novo* (4). In this respect, *Adnp*^+/−^ mice suffer from learning and memory deficiencies, muscle weakness, and communication problems. Data have shown the resolution of these symptoms with the administration of CP201, which also reduces neurodegeneration (20, 22). Mice with both *Adnp* genes deleted (*Adnp*^−/−^) do not survive, as Adnp is critical for neural tube closure and further brain formation (4, 23). Most recent data showed direct protection of CP201 against deleterious effects of ADNP pathogenic sequence variants spanning the ADNP protein (24, 25) as detailed below.

### Symptoms

Children are delivered on time (normal length and weight) (1, 26). Dysmorphic facial features are common, including a prominent forehead, high hairline, widely spaced and down-sloping eyes, posteriorly rotated ears, large head, long flat philtrum, thin upper lip, and a flat/broad nasal bridge ((4); https://www.adnpfoundation.org/). Other symptoms include seizures, hypotonia, feeding difficulties, gastroesophageal reflux disease, constipation, vomiting, heart defects (atrial septal defects and mitral valve prolapse), brain abnormalities (anxiety, aggressiveness, obsessive compulsive disorder), delayed milestones, severe cognitive delays, language disorder, motor skill disorder, undescended testicles, bilateral cryptorchidism, congenital hernia, and visual disturbances (hypermetropia, strabismus, and ptosis) (1, 5, 26). The main, similar features include gross and fine motor delay, along with intellectual disability (ID) and speech delay (10, 26–28).

Musculoskeletal defects have also been noted. These include joint hyperlaxity and multiple hand abnormalities, including, but not limited, to clinodactyly and abnormal phalanges (1). These children are also plagued with recurrent infections of the upper respiratory and urinary tracts (1). Abnormalities seen on brain magnetic resonance imaging (MRI) include wide ventricles, white matter lesions, and choroid cysts (1).

### Diagnosis

Diagnosis is usually made by identifying a heterozygous *ADNP* mutation through molecular genetic testing using whole-exome sequencing (4, 5, 10). Other molecular testing approaches are acceptable including single-gene testing and multigene panels (4). Commonly, the mutation is a *de novo* mutation, meaning it is a spontaneous pathogenic sequence variant within the DNA (5, 10).

Early tooth eruption is a common trait found in these children (6). Usually, by the end of their first birthday, the children have a full mouth of teeth including their molars. This premature teething can be an early diagnostic marker for *ADNP* mutations, which can pave the way to early intervention and personalized treatment (6).

## Standard of Care

Currently, there is no cure for this disease, and the prognosis for this syndrome is unknown (26). Although there is no standard of care for these children, they are symptomatically treated with walkers and surgically treated for atrial septal defects, ventricular septal defects, cardiovascular valve prolapse, imperforate anus, and astigmatism, along with other anatomical defects (10). Occasional treatments with risperidone have also been reported. Specifically, one case study on a 2½-year-old patient described that application of antipsychotic medication resulted in a significant resolution of behavioral outbursts, leading to progress in language acquisition (29).

Additional current treatments include physical therapy, occupational therapy, behavioral therapy, sensory processing therapy, and music and water therapy. Improvement with therapeutic intervention would prove beneficial to these patients and to caregivers (5, 10).

### Rationale for Drug Development

*ADNP* syndrome is a chronically debilitating disease to which there is no approved treatment. Although current pharmacological treatments can be effective at treating children symptomatically, a treatment to help with ID could potentially be life changing. Usually, treatment of these children involves multiple specialists that include neurologists, cardiologists, and surgeons (https://www.orpha.net/consor/cgi-bin/OC_Exp.php?Lng=EN&Expert=404448; https://rarediseases.info.nih.gov/diseases/12931/adnp-syndrome) (26).

This disease affects not only the child, but also the parents and the health care system. In the first few years of life, a child may go through multiple surgeries, including open heart surgery with ADNP involved in heart development (13) and affecting congenital heart diseases (5, 30). The cognitive impairments (ID) may be very severe; a 7-year-old may behave like a 16-month-old, and the language of a 3-year-old may be equivalent to a 12-month-old, as words are unrecognizable (5, 10, 31).

Looking at more than anecdotal case highlights, an extensive worldwide cohort of 78 individuals with *ADNP* syndrome was collected (2014–2016). The comprehensive results are published including clinician and parental interviews (26). In summary, clinical features include ID, autistic traits, severe motor and language development delays, and common facial appearances (outlined above). Behavioral problems, sleep irregularities, epilepsy, visual problems, hypotonia, congenital heart defects, gastrointestinal irregularities, short stature, endocrine (hormonal) deficiencies, and brain abnormalities (MRI) were described as common comorbidities. All these emphasize a need for further drug development.

Although rare, there have been at least two cases of childhood deaths (personal Facebook, https://www.facebook.com/TeamKnoxJoseph/; https://www.adnpkids.com/adnp-angels.html) with one recently published (25).

Postmortem analysis was conduct on a 7-year-old boy, heterozygous for *ADNP de novo* pathogenic sequence variant c.2244Adup/p.His559Glnfs^*^3. The child had autism, motor delays, severe ID, and seizures. He died following liver transplantation and multiple organ failure. A comparison to young adult with no tauopathy emphasizes the disease severity (25). Thus, a widespread child brain tauopathy paralleled by extensive transcriptomic alternations was discovered. Tauopathy was explained by direct ADNP mutation inhibition of Tau–MT binding (25). As tauopathy is a progressive condition, treatments halting tauopathy progression are required (24).

Therefore, the *ADNP* syndrome, in some cases, may be a devastating disease that does not allow children suffering from this disease to integrate into society due to multiple serious medical problems such as feeding difficulties, developmental delays (memory loss, limited speech), anatomical defects, and limited mobility.

To this end, CP201 is being developed for the treatment of the *ADNP* syndrome. This is based on reports of CP201 administration in heterozygous (haploinsufficient) mouse models of ADNP that has shown amelioration of some cognitive abnormalities along with restoration of learning and memory, skeletal strength, and vocalization with a reduction in neurodegeneration (22, 32–36). This is further based on CP201 mechanism of action as illustrated below.

## Drug Candidate: CP201 (NAP) Mechanism of Action

ADNP is critical for the brain, influencing brain development, brain injury protection, and aging. ADNP has been associated with EB1/EB3 (end-binding proteins) through the CP201 active motif mediating MT neuroplasticity (37). ADNP deficiency in mice impedes axonal transport (6, 38). Neuronal communication depends on MT integrity, and disruption results in delayed cognition. CP201 is brain bioavailable, benefiting synaptic development by promoting neuronal cell survival, synaptic maturation, neuroplasticity, and axonal transport. Alternative names include AL-108, NAP, NAPVSIPQ (molecular weight, 824.9 Da), and davunetide. CP201 is an intranasal (IN) investigational drug product constituting a multidispensing, metered nasal spray pump device including an aqueous solution of davunetide. It is packaged in a mechanical multi-dose device designed for the IN application of solutions.

Specifically, CP201 exhibits brain bioavailability (39, 40) and cellular bioavailability (41). The mechanism of action of CP201 is through its interaction with the MT EB-interacting motif (SxIP) in ADNP (binding to the neuroactive proteins EB1 and EB3) (37). CP201 is shown to enhance ADNP-EB1/EB3/Tau interaction (6, 42, 43) even in the face of ADNP mutations (24, 25).

By binding to EB1/EB3 and promoting other SxIP-containing proteins including ADNP to associate with EBs, CP201 also enhances MT impact on neuroplasticity and neuroprotection (37). Furthermore, by binding CP201 and EB1/EB3, the Tau–MT interaction is dramatically increased leading to neuron/brain protection (6). As such, CP201 promotes formation of mature dendritic spines (post synapse) (17, 22), enhances MT invasion to the tip of the growth cone (pre-synapse) (33, 44) and protects MT-dependent axonal transport (6, 38). This explains the breadth and efficiency of CP201's neuroprotective capability along with its neurotrophic capacities (6, 37). Heterozygous mutations of *Adnp* (*Adnp*^+/−^) result in Tau (MT associated protein) hyperphosphorylation paralleled by cognitive deficits. CP201 enhances Tau–MT binding and inhibits Tau hyperphosphorylation and aggregation, therefore, reversing ADNP deficiency (21).

Specifically, cellular expression of heterozygous ADNP truncating mutations (representing the majority of the *ADNP* syndrome cases, e.g., ADNP p.Ser404^*^ or p.Tyr719^*^, or p.Arg730^*^) reduced Tau–MT interactions (25) and impaired MT dynamics, in cell culture models (24). We have previously shown that CP201 enhanced the interaction of the intact ADNP with MT-Tau (6, 37). Thus, treating the ADNP-mutated cells with CP201 protected against ADNP mutation-induced MT dysfunction (24, 25). These results suggest that CP201-induces increased interaction of the intact ADNP with MT-Tau (6) and provides cellular protection (37), in the face of ADNP pathogenic sequence variants (25).

Furthermore, autophagy, a major cellular regulatory mechanism, is dependent on MTs, and ADNP binding to the MT associated protein 1 light chain 3 (LC3) is enhanced by CP201, protecting autophagy (45).

We propose CP201 as a first-in-class drug candidate, leading to the discovery of new routes to combat devastating brain diseases associated with the loss of essential cellular functions that culminate in loss of crucial daily functions (37).

### Preclinical Studies

Toxicology and pharmacology studies in animals were conducted with davunetide, the Drug Substance (DS). The DS used was of similar purity and quality as the batch used to produce the clinical supplies described here. For intravenous (IV) administration in the non-clinical acute dog toxicity study and the safety pharmacology studies, davunetide was dissolved in sodium chloride for injection. For IN administration in the non-clinical toxicity studies, davunetide was dissolved either in sodium chloride for injection or in a solution containing 7.5 mg/mL sodium chloride, 1.7 mg/mL citric acid monohydrate, 3 mg/mL disodium phosphate dihydrate, and 0.01% benzalkonium chloride. The IN toxicology studies used the same formulation composition as for the davunetide clinical supplies, except that the concentration of benzalkonium chloride used in the toxicology studies was twice the concentration in the clinical formulation (0.01% for animal studies vs. 0.005% for clinical supplies). Benzalkonium chloride is used as a preservative or bacterial-static agent commonly found in IN drugs. Proof-of-concept studies are described below.

The safety of davunetide was studied in various modes of administration (IN or IV) in a broad spectrum of doses (up to 300 mg/kg per day) and in several animal model (rats, dogs, and mice), as well as studies in juvenile animal performed in 6-week-old rats and 4–5-month old beagle dogs. The studies included safety pharmacology, acute dose toxicity, repeat dose toxicity in various lengths and designs, genotoxicity, pharmacokinetic analysis, and drug–drug interaction where the inhibition of CYPs was studied. The product was well tolerated in the non-clinical studies and did not demonstrate any test article related adverse events (39, 40). Davunetide demonstrated a maximal NOAEL of 20 mg/kg per day in IN administration in dogs, which is equivalent to 11.11 mg/kg per day in humans.

### Proof of Concept Studies

#### *In vitro* 

CP201 (NAP) was extensively studied in multiple *in vitro* studies. A previous review summarized the pharmacology up to 2017 (46). Here, *in vitro* studies related to the mechanism of ADNP are summarized in Table 1. These studies demonstrate that CP201 directly affects ADNP mechanisms. In neuronal cell cultures, CP201 increased dendritic spine plasticity and protected neurons through the SxIP motif, while enhancing endogenous ADNP interaction with MTs and Tau.

**Table 1:** *In vitro* preclinical proof-of-concept studies.

**Study title**	**Purpose**	**Assay and concentrations**	**Results**	**Conclusion**
The CP201 motif of ADNP regulates dendritic spines through microtubule end binding proteins (37)	To evaluate the requirement of the SxIP motif (microtubule interacting motif) in CP201 and ADNP in the modulation of synaptic plasticity and cell protection	Primary neurons, COS7 cells, PC12 cells (CP201, 10^−15^-10^−9^ M) Measurements of protein characteristics for dendritic spines. Immunochemistry, immunopercipitation EBs RNA silencing Affinity chromatography and cell survival assays	CP201 increased dendritic spine plasticity and protected neurons through the SxIP motif, while enhancing endogenous ADNP interaction with microtubules	The identified SxIP shared by CP201/ADNP is directly implicated in synaptic plasticity, explaining the wide scope and potency of neurotrophic/neuroprotective capacities
ADNP/CP201 dramatically increase microtubule end-binding protein-Tau interaction: a novel avenue for protection against tauopathy (6) even in the face of multiple ADNP mutations (24, 25)	To evaluate the effect of CP201 on Tau–microtubule interaction through the SxIP motif	N1E-115 neuroblastoma neuronal cell model. Immunochemistry, cell transfection with fluorescent proteins, and live cell imaging. Mutations tested include ADNP-p.Ser404*, p.Tyr719*, and p.Arg730*. NIH3T3 fibroblasts, cell transfection with Tau and live cell imaging; immunopercipitation (CP201, 10^−12^ M)	CP201 augmented microtubule dynamics in N1E-115 neuroblastoma neuronal model. CP201 dramatically increased Tau–microtubule interaction through its SxIP motif and protected NIH3T3 cells against zinc intoxication, only if these cells were transfected with Tau	Microtubule–Tau binding is identified as a new site for endogenous ADNP neuroprotection, and a target for drug development, with CP201 as a lead compound
Premature primary tooth eruption in cognitive/motor-delayed ADNP-mutated children and activity-dependent neuroprotective protein deficiency models synaptic and developmental phenotypes of autism-like syndrome (6, 22, 25, 47)	To compare gene expression patterns in ADNP patient-derived lymphoblastoid cells (LCLs) to *Adnp^+/−^* mouse hippocampal, cortical, and splenic expression levels and evaluate protection by CP201	Cellular mutations tested: ADNP-p.Arg216*, ADNP-p.Lys408Valfs*31, and ADNP-p.Tyr719* CP201 was administered *in vivo* (intranasal) at 0.5 μg/mouse, 1 month of daily intranasal administrations (starting at 2 months of age)	1,442 common genes were differentially expressed in all three different ADNP-mutated cell lines compared to the control cell line. RNA transcripts changed by *ADNP* deficiency and reversed by CP201 treatment in the mouse spleen were also found to be changed by various human ADNP mutations in the ADNP-mutated cells	Tested *ADNP* syndrome pathogenic mutations cause loss of function (ADNP haploinsufficiency). Gene expression patterns affected by ADNP loss of function are partially ameliorated by CP201 treatment
Cellular and animal models of skin alterations in the autism-related *ADNP* syndrome (32).	Test the involvement of ADNP in skin function and CP201 ameliorative effects on dermal thickness and wound healing	ADNP Tyr719* patient-derived skin cells, 100 or 600 nM CP201 or mouse *Adnp^+/−^* fibroblasts, 180 nM CP201	Ameliorative effects of drug treatment on skin abnormalities, specifically wound healing, which seems to be impaired (see also *in vivo* results, Table 2)	A new activity of ADNP was discovered in the skin that may serve to characterize the clinical phenotype of patients with *ADNP* syndrome. The study further provides a therapeutic option for skin deficits in these patients

#### *In vivo* 

##### The Adnp^+/−^ Mouse Model

The *Adnp*^+/−^ mouse model predicted the *ADNP* syndrome (20). This mouse line exhibits developmental delays and synaptic dysfunctions mimicking children with *ADNP* syndrome. A survey of 78 children carrying pathogenic *ADNP* sequence variants spanning the entire protein suggested partial loss of similar functions, with potentially some increased severity in ADNP p.Tyr719^*^ children (the most prevalent group) (26). Importantly, the mutated (mostly truncated) and the intact *ADNP* alleles are both expressed in *ADNP* syndrome human cells (4), supporting the *Adnp*^+/−^ mouse as a model predictive for *ADNP* heterozygous mutation deficiency in humans (6, 26). Furthermore, some children with *ADNP* syndrome show almost complete deletions of one allele (9), presenting a haploinsufficient loss-of-function phenotype (1, 9). Finally, there is a very high conservation of the *ADNP* gene between human and mouse (about 90% identity at the mRNA level) (8), and ADNP is critical for brain development in the mouse, like in human (23).

The *Adnp*^+/−^ mouse model is representative of traits presented in children with *ADNP* syndrome as described before (22). The protective effect of CP201 was demonstrated by affecting animal traits that are equivalent to clinical symptoms in human patients with *ADNP* syndrome (20, 22). A summary of *in vivo* proof-of-concept studies in the *Adnp*^+/−^ mouse model is provided in Table 2 and expended below.

**Table 2:** *In vivo* preclinical proof-of-concept studies.

**Study title**	**Purpose**	**Animal model**	**Study description**	**Results**	**Conclusion**
The ADNP snippet NAP reduces Tau hyperphosphorylation and enhances learning in a novel transgenic mouse model (20)	To generate a transgenic mouse devoid of one *Adnp* allele and to assess CP201 activity *in vivo*	*Adnp/^+/−^* mice (*Adnp*^−/−^ was embryonic lethal) (23)	Newborn mice administered daily with SC CP201 (25–500 μg/kg) and subjected to behavioral testing. In a separate experiment, CP201 was administered IN daily over a 2-week period to 2- and 9-month-old male mice (0.5 μg/5 μL/mouse per day.	*Adnp^+/−^* mice exhibited cognitive deficits, significant increases in pathological phosphorylated Tau compared with *Adnp^+^*^/+^ mice. CP201 treatment partially ameliorated cognitive deficits and reduced Tau hyperphosphorylation in the *Adnp^+/−^* mice	These results imply that ADNP is critical for brain activity, participating in normal cognitive function. *Adnp*-deficient mice were shown to be a model for evaluation of cognitive enhancers, such as CP201, which ameliorated cognitive deficits associated with ADNP deficiency
ADNP is an alcohol-responsive gene and negative regulator of alcohol consumption in female mice (35)	To assess the ADNP/CP201 role in the regulation of alcohol consumption	*Adnp*^+/+^ and littermates, *Adnp*^+/−^ mice (outbred with the ICR strain for 30 generations) (21)	*Adnp* ^+/−^ or *Adnp*^+/+^ mice (25–30 g, *n* = 14–15/experimental group) were given continuous access to two bottles: water and 10% alcohol, for 4 weeks. After 1 week of drinking without treatment, all mice received vehicle treatment for 1 week, followed by intranasal CP201 (0.5 μg/5 μL) treatment for 2 weeks, 5 days per week	The *Adnp^+/−^* female mice showed higher alcohol consumption and preference, compared to *Adnp^+/−^* females. Daily intranasal administration of CP201 normalized alcohol consumption in the *Adnp^+/−^* females	ADNP is a potential new biomarker and regulator of alcohol-drinking behaviors. CP201 corrected the phenotype, suggestive of corrected obsessive/addictive phenotype
Activity-dependent neuroprotective protein deficiency models synaptic and developmental phenotypes of autism-like syndrome (22)	The study was conducted to correlate one-to-one the children phenotype to the *Adnp^+/−^* mouse phenotype, and to assess CP201 protection and target engagement	A unique neuronal membrane tagged GFP-expressing *Adnp^+/−^* mouse line allowing *in vivo* synaptic pathology quantification	For dendritic spine determinations, 3-month-old *Adnp*-GFP mice were treated for 9 consecutive days with either intraperitoneal CP201 injection (0.4 μg) diluted in 0.1 mL saline or with 0.1 mL saline as vehicle. On day 9, mice were perfused, and brains were subjected to immunohistochemistry	ADNP deficiency reduced dendritic spine density and altered synaptic gene expression, both of which were partly ameliorated by CP201 treatment. *Adnp^+/−^* mice further exhibited global developmental delays, vocalization impediments, gait/motor dysfunctions, and social/object memory impairment, all partially reversed by daily CP201 administration (systemic/intranasal)	This study associated ADNP-related synaptic pathology to developmental/behavioral functions, establishing CP201 *in vivo* target engagement. The study further identified potential future biomarkers. The results of the study provide incentive to clinical development of CP201 in the *ADNP* syndrome
Hanging wire test: *Adnp*^+/−^ mice display decreased latency to fall in an age- and sex-dependent manner—NAP protects (22, 47)	To assess the effect of CP201 on reduced grip strength	*Adnp*^+/+^ and *Adnp*^+/−^ mice	2-month-old mice (*n* = 3–4 males or 6–8 females per experimental group) were treated daily, five times a week for 5 weeks with 0.5 μg NAP/mouse per day by intranasal administration. Grip strength was measured by hanging wire tests	The time it took *Adnp^+/−^* CP201-treated mice to fall off the inverted cage lid was 90 s, similar to the time it took the *Adnp*^+/+^ mice to fall off, as opposed to ~15 s for the *Adnp^+/−^* mice. Sexual dichotomy was also observed in *Adnp^+/−^* mice (*p* < 0.05)	Male *Adnp^+/−^* mice exhibited decreased latency to fall, as compared to *Adnp*^+/+^ mice, which was improved by CP201 administration
Grip strength test: *Adnp* mice exhibit significant decreased grip force—NAP protects (22, 47)	To assess the effect of CP201 on reduced grip strength	*Adnp*^+/+^ and *Adnp*^+/−^ mice	Two-month-old mice were treated by daily intranasal administration of 0.5 μg CP201/mouse, five times a week for 5 weeks. Grip strength was measured by using the Ugo Basile 47200-Grip-Strength Meter	*Adnp^+/−^* mice demonstrated decreased strength for males and females as opposed to the strength displayed by the male and female *Adnp*^+/+^. The treatment of CP201 restored the grip strength of the *Adnp^+/−^* mice for males and females, respectively. Sexual dichotomy was also observed in *Adnp^+/−^* mice (*p* < 0.05)	*Adnp^+/−^* male mice exhibited reduced muscle strength vs. *Adnp*^+/+^ mice, with CP201 significantly improving it.
NAP treatment protected against vocalization deficiency in *Adnp^+/−^* mice (22, 47)	To assess the effect of CP201 on speech deficits	*Adnp*^+/+^ and *Adnp*^+/−^ mice	Ultrasonic vocalizations (USVs) were recorded in 8-day-old pups, subjected to daily subcutaneous injections of NAP (25 μg/mL saline) or saline (20 and 40 μL on postnatal days 1–4 and 5–7).	The *Adnp^+/−^* mice had a decrease in the number of vocalizations per minute at approximately ½ the number seen in the *Adnp*^+/+^ mice. When the *Adnp^+/−^* mice were treated with CP201, the number of vocalizations increased to over 18 vocalizations per minute	CP201 administration increased vocalization in the *Adnp^+/−^* mice, suggesting that CP201 has the potential to treat vocal communication deficits
Cellular and animal models of skin alterations in the autism-related *ADNP* syndrome (32)	Test the participation of ADNP in skin function and CP201 ameliorative effects on dermal thickness and wound healing	*Adnp*^+/+^ and *Adnp*^+/−^ mice. ADNP p.Tyr719* patient derived skin.	Sonography in the patient revealed thin skin. Dermal thickness measurements in the mice in the presence and absence of CP201 treatment. Nasal CP201 application (0.5 μg CP201 in 5 μL vehicle solution) was performed daily, once a day, for 6 weeks (5 days a week). Vehicle-treated mice were maintained until the age of 4.5 months	The human and the *Adnp^+/−^* mice had thinner skin, which was normalized by CP201 treatment	The study discovered a new activity of the autism-linked ADNP in the skin. This activity may serve to define the clinical phenotype of patients with *ADNP* syndrome. Furthermore, the results suggest CP201 as an attractive medication for skin problems in ADNP patients
Microbiota changes associated with ADNP deficiencies: rapid indicators for NAP (CP201) treatment of the *ADNP* syndrome and beyond (36)	As the microbiome interacts with brain function, we investigated the effects of the *Adnp^+/−^* genotype on microbiota composition in our *Adnp^+/−^* mouse model	*Adnp*^+/+^ and *Adnp*^+/−^ mice (1-month-old on the first treatment day)	DNA obtained from fecal bacterial loads was subjected to PCR to identify different microbiota with and without CP201 treatment (nasal application 0.5 μg/5 μL/mouse per dose, daily for 45 days)	A highly significant sexually dichotomized *Adnp* genotype effect and amelioration by CP201 was observed as described below. Most of the commensal bacterial microbiota tested were affected by the *Adnp* genotype and corrected by CP201 treatment in a male sex-dependent manner. A female *Adnp^+/−^* genotype linked decrease (contrasting with a male increase) was observed in the *Lactobacillus* group. Significant correlations were found between specific bacterial group loads and behavior in the open-field and the three-chamber apparatus measuring social behavior.	ADNP deficiency–associated changes in commensal gut microbiota compositions and a sex-dependent biomarker for the *ADNP* syndrome were discovered. Strikingly, a rapidly detected CP201 treatment-dependent biomarkers within the gut microbiota was also discovered. Because gut microbiota are closely associated with immune responses, and CP201 modulates the immune response toward an anti-inflammatory response (48, 49), microbiota and immune markers are now under patent protection (Ramot at Tel Aviv University)
Age- and sex-dependent ADNP regulation of muscle gene expression is correlated with motor behavior: possible feedback mechanism with PACAP (16)	Understand the involvement of ADNP and CP201 in muscle transcriptomic patterns, in correlation with motor activity throughout the entire life span	*Adnp*^+/+^ and *Adnp*^+/−^ mice	Using quantitative RT-PCR, the *Adnp*^+/−^ genotype in mice resulted in aberrant gastrocnemius muscle, tongue and bladder mRNA transcript expression, which was ameliorated by CP201 treatment.	A significant sexual dichotomy was revealed, coupled to muscle-, and age-specific transcriptional regulation. *Adnp*/CP201 regulated myosin light chain (*Myl*) in the gastrocnemius muscle, the language acquisition gene forkhead box protein P2 (*Foxp2*) in the tongue and the bladder-function linked, pituitary–adenylate cyclase activating polypeptide (PACAP) receptor PAC1 mRNA (*Adcyap1r1*) in the bladder. A significant age dependency was discovered, coupled to an extensive correlation to muscle activity (gait)	The results suggest a tight connection between Adnp and muscle activity throughout life, including (1) the acto-myosin muscle system (Myl2 and Myl9), (2) energy metabolism nicotinamide nucleotide adenylyl (NAD) transferase 1 (Nmnat1) (50), (3) speech acquisition Foxp1/Foxp2 tongue expression, (4) bladder activity feedback regulation (PACAP), and (5) multiple correlations with gait functions. Sexual dichotomy provides guidelines for better clinical design

### ADNP Deficiency in Mice Models the ADNP Syndrome

Results comparing synaptogenesis, dendritic spine formation, and immunohistochemistry of excitatory synapses in the *Adnp*^+/−^ mice to human *ADNP* syndrome MRI data have been collected (22, 33, 34). These results demonstrate parallels between the *Adnp*^+/−^ mouse and patients with *ADNP* syndrome, at multiple levels (developmental, behavioral, and motor). Furthermore, the mouse model allowed quantitation of excitatory synapse density in the hippocampus and motor cortex and evaluation of transcriptomic data, correlating molecular, anatomical, and functional consequences as described (22). These results establish CP201 *in vivo* target engagement and identify potential biomarkers, paving the way toward clinically advancing CP201 for the *ADNP* syndrome.

The data in *Adnp*^+/−^ mice further demonstrate that hyperphosphorylation of Tau is decreased following CP201 treatment (20). This is in line with the findings of tauopathy in the human postmortem ADNP case and with mutated human ADNP reducing Tau–MT interaction, which is corrected/normalized by CP201 treatment (25).

Collectively, the data from *ADNP* syndrome mouse model demonstrate CP201 to be a promising therapeutic candidate for the treatment of children who suffer from this debilitating disease (Table 2).

It should be added that although the current review may seem limited in cellular and animal models, a previous book chapter summarized CP201 (NAP) *in vitro* and *in vivo* pharmacology up to 2017. This previous report includes dozens of our own investigations, as well as independent research in versatile disease models corroborating the proposed efficacious mechanism of action (46).

## Clinical Studies

CP201 has not been previously approved for the treatment of the ADNP syndrome; however, clinical trials for other indications have been conducted [progressive supranuclear palsy (PSP), mild cognitive impairment (MCI), and schizophrenia] (42).

CP201 was previously referred to as AL-108, developed by Allon Therapeutics and subsequently licensed by Coronis Neurosciences from Ramot at Tel Aviv University.

The legal owner of all Allon Therapeutics materials is Ramot. Previous clinical trials for IN administered davunetide by Allon include the following:

1. ClinicalTrials.gov identifier: NCT00422981—MCI
2. ClinicalTrials.gov identifier: NCT00505765—Schizophrenia
3. ClinicalTrials.gov identifier: NCT01056965—Tauopathies
4. ClinicalTrials.gov identifier: NCT01110720—PSP

Allon also conducted an IV administration trial:

ClinicalTrials.gov identifier: NCT00404014—MCI Following Coronary Artery Bypass Graft Surgery.

No significant side effects were reported. Minor side effects in a small minority of patients may have included some nasal discomfort (51), which could perhaps be associated with the application volume requiring repeated daily nasal administrations (52). In general, all studies have proven safety and tolerance of CP201 in hundreds of adult compromised patients. Efficacy was seen in enhancement of cognitive function and functional activities of daily living as reviewed (46).

Additional clinical studies have shown that ADNP levels correlate with disease status (cognitive impairments, and schizophrenia) and tauopathy as illustrated above, e.g., ClinicalTrials.gov identifier: NCT01403519—Innovative Biomarkers in Alzheimer's Disease and Frontotemporal Dementia: Preventative and Personalized (24, 53).

Current *ADNP* syndrome clinical trials feature natural history (e.g., ClinicalTrials.gov identifier: NCT01238250 and NCT03718936). Furthermore, ketamine is being tested in the *ADNP* syndrome patients ClinicalTrials.gov identifier: NCT04388774, and as noted above, risperidone treatment has shown some efficacy in a case study (29).

Coronis was granted an Orphan Drug Designation #DRU-2017-6243 by the US Food and Drug Administration (FDA) for the treatment of the *ADNP* syndrome with CP201. Coronis has further officially met with the FDA for a Pre-Investigational New Drug Application, paving the path to a CP201 clinical trial (54).

## Author Contributions

IG designed, led and orchestrated the writing, provided funding, designed experiments, analyzed the data of many of the cited articles, and wrote the final mini review.

## Conflict of Interest

IG is the Chief Scientific Officer of Coronis Neurosciences. NAP (CP201) use is under patent protection (US patent nos. US7960334, US8618043, and USWO2017130190A1).
